# Causal mechanisms of postnatal depression among women in Gondar town, Ethiopia: application of a stress-process model with generalized structural equation modeling

**DOI:** 10.1186/s12978-020-00912-z

**Published:** 2020-05-07

**Authors:** Abel Fekadu Dadi, Lillian Mwanri, Richard J. Woodman, Telake Azale, Emma R. Miller

**Affiliations:** 1 Department of Epidemiology and Biostatistics, Institute of Public Health, College of Medicine and Health Sciences, University of Gondar, Gondar, Ethiopia; 2 Flinders University, College of Medicine and Public Health, Health Sciences Building, Sturt Road, Bedford Park, Adelaide, 5001 SA Australia; 3 Flinders University, College of Medicine and Public health, Center for Epidemiology and Biostatistics, Health Sciences Building, Sturt Road, Bedford Park, Adelaide, 5001 SA Australia; 4 Department of Health Promotion and Behavioral Sciences, Institute of Public Health, College of Medicine and Health Sciences, University of Gondar, Gondar, Ethiopia

**Keywords:** Postnatal depression, Low birth weight, Self-reported labor complication, Ethiopia

## Abstract

**Background:**

Postnatal depression (PND) is the second most common cause of disability and the most common complication after childbirth. Understanding the potential mechanisms by which the stress process can lead to PND is an important step for planning preventive interventions for PND. This study employed a stress process model to explore the possible pathways leading to PND in Gondar Town, Ethiopia.

**Methods:**

A community-based cohort study was conducted in 916 pregnant women, who were assessed for depression in their second or third trimester of pregnancy and re-assessed two to eight weeks after birth. Women with an Edinburgh Postnatal Depression Scale (EPDS) ≥6 were considered to be depressed. Modified Poisson regression was used to identify the independent predictors of PND. A Generalized Structural Equation Modeling (GSEM) was then used to explore the direct and indirect effects of stressors and their mediators on PND.

**Results:**

The prevalence and incidence proportion of PND were 9.27% (95%CI: 7.45, 11.36) and 7.77% (95%CI: 6.04, 9.79), respectively and 2.1% of the women demonstrated symptoms of depression within the study period. PND was independently predicted by having limited postnatal care services, Antenatal Depression (AND) and a Common Mental Disorders (CMD) before pregnancy, (IRR = 1.8; 95%CI: 1.0, 3.2), 1.6(95%CI: 1.4, 1.7), and 2.4 (95%CI: 1.4, 4.3) respectively). In SEM, AND (standardized total effect = 0.36) and a CMD before pregnancy (standardized total effect = 0.11) had both a direct and an indirect positive effect on PND scores. Low birth weight (standardized β = 0.32) and self-reported labor complications (standardized β = 0.09) had direct effects only on PND scores.

**Conclusion:**

The observed incidence and prevalence of PND in Ethiopia were lower than in previous studies. A CMD before pregnancy and low birth weight (LBW) increased PND scores, and these effects were in part mediated via antenatal depression and labor complications. Early detection and treatment of depression before or during pregnancy could either directly or indirectly reduce the risk of labor complications and PND. Interventions that reduce LBW or improve the uptake of postnatal care might reduce PND incidence.

## Plain English summary

### Background

Postnatal depression (PND) is the second most common cause of disability and the most common complication after childbirth. Understanding the potential mechanisms by which stress processes can lead to PND is an important step for planning PND preventive interventions. This study explored the possible causal mechanisms leading to PND in Gondar Town, Ethiopia. A community-based cohort study was conducted by recruiting 916 pregnant women who were assessed for depression in their second or third trimester of pregnancy and re-assessed two to eight weeks after birth. Women with an Edinburgh Postnatal Depression Scale (EPDS) ≥6 were considered as depressed.

The prevalence and incidence of PND were 9.27 and 7.77%, respectively, and 2.1% of the women had symptoms of depression within the study period. PND was independently predicted by having no postnatal care services, having Antenatal Depression (AND) and Common Mental Disorders (CMD) before pregnancy. Antenatal depression and a CMD before pregnancy had both a direct and an indirect positive effect on PND scores. Low birth weight (LBW) and self-reported labor complications had direct effects only on PND scores. The observed incidence and prevalence of PND in Ethiopia were lower than in previous studies. A CMD before pregnancy and LBW increased PND scores, and these effects were in part mediated via antenatal depression and labor complications. Early detection and treatment of depression before or during pregnancy could either directly or indirectly reduce the risk of labor complications and PND. Interventions that reduce LBW or improve the uptake of postnatal care might reduce PND incidence.

## Background

Postnatal depression (PND) manifests within four to six weeks after birth [1], reaches a peak prevalence at two to three months [2–4] and can persist for one year. Social construct causes of postnatal depression include poor relationships, and difficulty with pregnancy and caring for the baby [5].

PND is the leading cause of disability [6] and the most common complication of childbirth [7] worldwide. PND increases costs to the health care system and reduces the workforce economy [8]. It also reduces the maternal quality of life [9] due to higher risk of back pain, reduced feelings of self-worth, increased thoughts of self-harm and suicidal ideation [10], insomnia, negative parenting behavior and in extreme cases the risk of committing infanticide [11]. Depressed women have been reported to struggle with efficient breastfeeding; use available health services less frequently [11, 12]; have negative postnatal birth experiences [13]; develop anemia during pregnancy [14]; experience preterm birth and babies with low birth weight; have ongoing infant illness/disability; receive low social support [15, 16]; be at risk for use substance misuse [17]; tend to be less engaged in of physical activity [18]. PND negatively affect maternal emotional regulation, stress coping capability, interaction with their newborn [19], and child’s cognitive development through impairing maternal mental and behavioral care [20].

A review compiled from 53 low and middle-income countries reported a pooled postnatal depression prevalence of 19% [21]. Two African systematic reviews reported a pooled prevalence of 18.3% [22]*,* which is as high as those reported in other developed countries [23]. Recent Ethiopian studies have reported PND prevalences to range from 12.2 to 22.1% in rural areas [24, 25] and 22.4 to 33.2% [26–28] in urban areas. However, none of these studies has reported PND in Amhara region where the current study was conducted.

A review of quantitative and qualitative studies conducted in Sub-Saharan Africa reported that stressful life events, negative effects of cultural perception and practices, and the presence of extended family as factors contributing to the occurrence of postnatal depression [29]. Though the extended family is not an issue in Ethiopia, non-specified cultural perceptions and practices would be the most important factors of PND, specifically in Gondar. Some limitations of recently published studies may include measurement error due to the inclusion of postnatal mothers in their first fifteen days of delivery [26–28], focusing in a single region, omitting consideration of important confounding factors of PND such as antenatal depression, birth outcomes, postnatal care and labor complications [24, 26–28].

Pearlin and his colleagues argue that every common mental disorder should be presented in the form of a model that most explain how the stressors or risk factors cause the disorder [30]. They suggested a stress process model as a theoretical framework to be applied by studies interested in portraying causal pathways for common mental disorders. The stress process model consists of three main conceptual domains. The first domain consists of a source of stress (stressor) domain, which includes life events and chronic life strains. The second domain consists of the mediator’s domain, which includes any mediators of stress that have an ability to mediate the impact of stressful situations such as social support and coping styles from the stressor. The third domain consists of the stress outcome, which is a manifestation of the stress of various mental disorders. This stress process model has been tested both in pregnant [31] and postnatal [32] populations in China for specific to antenatal and postnatal depression and achieved better fit.

**Fig. 1:**
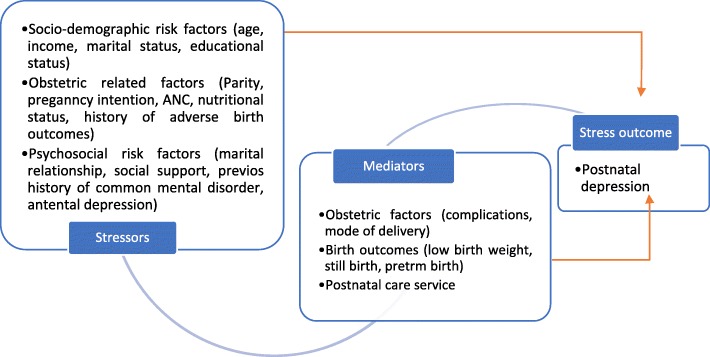
A hypothetical stress process model for postnatal depression based on Perlin et al., 1981

Understanding the potential mechanisms by which stresses can lead to depression is now considered essential to enable the development of preventative PND interventions [33, 34]. This study, therefore, considered the stress process model developed by Pearlin [30] to better understand potential causal pathways to postnatal depression among postnatal women in Gondar Town. We used structural equation modelling to fit the stress process model. We tested for its theoretical and statistical validity across the three stress process domains, namely: stressor, mediator, and stress outcomes. A hypothetical stress process model for postnatal depression is presented in Fig. 1.

## Methods

### Study design and setting

A community-based cohort study was conducted with pregnant women recruited in their second or third trimester of pregnancy. Participants were assessed at baseline and then re-assessed for postnatal depression two to eight weeks after childbirth in Gondar Town. Gondar town is one of the administrative zones of Amhara Regional State, Northwest Ethiopia. Gondar Town is 747 km away from Addis Ababa (the capital and largest city of Ethiopia), and it is mountainous area but accessible by any transportation facility. Gondar town has 12 kebeles (the smallest administrative units in the country) with a total population of 333,103 in 2017/2018. The number of pregnancies reported in 2017/2018 in Gondar town was 6450 [35, 36]. The town has one government-operated referral hospital, eight health centres, and 15 private medical clinics (Gondar Town Health office plan, 2017/18).

### Sample size

This analysis forms part of a larger mother-child health cohort study designed to examine the extent of perinatal depression and its effect on birth and infant health outcomes in Gondar Town. The required sample size was determined using an Epi Info version 7 [37] for the larger cohort study and used the following assumptions: a 95% confidence level, 90% power, an exposed to non-exposed (perinatal depression) ratio of 1:2, prevalence of underweight of 25%, and an odds of low birth weight for those exposed of 1.5. A sample size of 809 was determined with a further 20% to account for expected losses to follow up, extending the final sample size to 970. Ultimately, a sample of 960 pregnant women was recruited, and after excluding 44 pregnant women due to refusal, absenteeism and health issues, a total of 916 participants were followed to their postnatal period.

### Ethical approval

This project was approved by the Social and Behavioral Research Ethics Committee (SBREC) of the Flinders University [38] and the Institutional Review Board of the University of Gondar. A support letter was obtained from the Gondar town’s mayoral office and respective kebeles. Participants were informed about the purpose, objectives and their right to participate or withdraw from the research activities. After this, study participants were asked to sign the consent form and those illiterate were asked to use their thumb. Privacy and confidentiality were maintained throughout the study. Women and children who were found to be seriously ill and fulfilled the following criteria were referred to University of Gondar Specialized Hospital Psychiatry unit for further diagnosis and treatment: an overall Edinburgh Postnatal Depression Scale (EPDS) score of 13 and those who scored 1, 2, or 3 on item ten, which was a question about the thought of self-harm [39]. Those with an overall EPDS ≥17, indicating severe depression in need of care, were excluded from the study.

### Data collection and the questionnaire

Face-to-face interviews were conducted with postnatal women in their home Using a structured and pre-tested electronic-based questionnaire. Data collection was facilitated by using an online, Open Data collection Kit (ODK) application tool [40], which populated Microsoft Excel spreadsheets. These were checked for validity using Enketo (a preview provided by ODK). Nine trained nurse data collectors used Lenovo 7 tablets with uploaded questionnaires, and data were temporarily stored on the Google Cloud Platform. The principal investigator directly downloaded the data from the system to the project computer files, which were password protected. The questionnaire collected socio-demographic information such as age; sex; educational status (no formal education, grade 1–8, grade 9–12, diploma and above); income (low, medium, high); and marital status (single, married, separated). Information on maternal characteristics included pregnancy intention (planned, unplanned), gestation (weeks), previous history of adverse birth outcomes (low weight, preterm or stillbirth) and previous history of a caesarian section delivery. Finally, the questionnaire collected information on psychosocial and behavioral characteristics such as social support (good, poor), partner support (always, most of the time, some of the time, rarely), stress coping ability (very rarely, rarely, sometimes, most of the time), coffee drinking (daily, sometimes, never), and cigarette exposure (yes, no).

### Instruments

Postnatal depression was assessed using the Edinburgh Postnatal Depression Scale (EPDS), developed by Cox and colleagues [39], which was adapted for use in the Ethiopian context [41]. The tool is intended to measure the distress that pregnant women have experienced over the previous week [42–44]. The validity of EPDS for urban populations was tested in Addis Ababa with a sensitivity, specificity, and misclassification rate of 78.9, 75.3 and 24.0%, respectively at an optimal cut-off point of 6 [45]. Women were considered as having depression with an EPDS score of ≥6. Antenatal depression was also assessed by using the EPDS, and those with a score of ≥12 were considered as depressed during pregnancy. In the current study, the EPDS demonstrated high reliability for the single construct of depression with internal consistency (α) of 0.74. History of common mental disorders was assessed by a question ‘Have you experienced any symptoms of depression, anxiety or stress before your current pregnancy?’ (Yes, No).

Birth weight was obtained from medical records or, for those who did not give birth at a health facility (*n* = 38, 4.3%), measured at their home within 24 h of delivery using a digital SECA scale to the nearest 0.1 g. Low birth weight was classified as a birth weight less than 2500 g [46]. Gestational age was calculated based on the last normal menstrual period (LNMP) or from ultrasonography information obtained from the women. Preterm birth was considered as birth occurring before 37 completed weeks of gestation [46]. Stillbirth was viewed as the death of the fetus after 20 completed weeks of gestation or intrauterine death of the fetus prior to the onset of labor, or intrauterine death of the fetus during labor and delivery [47].

The Oslo Social Support Scale (OSSS-3) [48] was used to measure maternal social support during pregnancy, and it was assumed that this support was sustained after delivery. The OSSS-3 has three items measured by number of Likert scales, which are summed to 14 points and categorized as ‘poor’ (total score < 9) or moderate to strong (overall rating 9–14). In this study, the OSSS-3 demonstrated high reliability with internal consistency (α) of 0.76. Partner support was assessed by a question ‘My husband helps me a lot’ with five response scales, ‘Always’, ‘Most of the time’, ‘Some of the time’, ‘Rarely’, and ‘Never’. It was assumed that partner support during pregnancy would also be sustained after birth. The maternal Middle-Upper Arm Circumference (MUAC) tape was used to measure nutritional status. MUAC is validated and recommended tool for measuring nutritional status during the postnatal period, with a cutoff score of 18–22 considered as ‘normal’ and 22.5 to 31 as ‘underweight’ [49].

The marital agreement was assessed by the question ‘How often do you discuss and agree with your husband in day to day life? (Most of the time, Some of the time, Rarely, Never). The marital relationship was assessed by the question ‘How would you explain your marital condition in general?’ (Very good, Good, Bad, Very bad).

Pregnancy intention was assessed by the question ‘At the time you became pregnant with this pregnancy; did you want to become pregnant then, did you want to wait until later, or did you not want to have any more children? With response categories ‘Wanted now’, ‘Wanted later’, and ‘Not wanted at all’. The wanted now, or later options were combined and labelled as “planned” and ‘Not wanted at all’ as “unplanned”.

### Statistical analysis

Completed data were downloaded from the Google cloud platform in excel form, checked for completeness and imported to Stata version 14 (StataCorp, USA) for further cleaning and analysis. Descriptive analyses included identifying the mean, median, proportion/percentage, interquartile range, standard deviation. Exploratory analysis, such as crosstabs and frequencies, were conducted to understand the nature of the data. Chi-squared tests were used to test for crude associations between the stressors and risk of depression.

The main outcome of this analysis was postnatal depression, measured as a latent variable. Prior to fitting the SEM, a confirmatory factor analysis (CFA) was conducted to test the model fit of the Edinburgh Postnatal Depression Scale (EPDS) score. Indicators used to evaluate the SEM fit were the Sartorra-Bentler Chi-squared test of fit (*P* > 0.05), Comparative Fit Index (CFI ≥ 0.90), Tucker-Lewis Index (TLI ≥ 0.90), Root Mean Square Error of Approximation (RMSEA≤0.08), Standardized Root Mean Square Residual (SRMR≤0.08) and the coefficient of determination (R^2^) [50].

To fit the final structural model, variables were checked at a bi-variable level and those factors associated with postnatal depression at a *p*-value < 0.2 were further assessed in a multivariable mixed-effect linear regression model. This model was compared with a standard multivariate linear regression model using the likelihood ratio test [51] and was significant (*p* < 0.001). Variables significant (*p* < 0.05) in the multivariable mixed-effects linear regression were then used to build a structural model based on the stress-process framework.

A Generalized Structural Equation Modeling (GSEM) was used for the current analysis in order to handle both discrete and continuous endogenous variables [52]. GSEM allows for [1] evaluation of the potential causal pathways; [2] estimation of direct and indirect effects of multiple interacting variables and [3] appropriate use of different link functions and distributions [53, 54]. Mplus version 8.3 [55] was used for the GSEM and Stata version 14 was used for all other analysis.

## Result

A total of 916 participants were followed from their pregnancy for up to 45 days after birth. During this follow up period, four women refused to continue their participation and eight were lost from the study giving a lost to follow up rate of 1.3%. Postnatal depression screening was performed for 904 women during the period between 15 and 45 days after birth. Nine women had an EPDS≥17 and were excluded. A final group of 895 postnatal women were included in the analysis for postnatal depression prevalence, identification of predictors, and developing a stress-process SEM.

### Socio-demographic characteristics of study participants

The socio-demographic characteristics of the study participants are shown in Table 1. The mean (SD) age of study participants was 26.5 (0.15) and the majority (*n* = 639, 71.4%) were responsible for home duties, were Orthodox Christians (*n* = 722, 80.7%), and were partnered (*n* = 860, 96.1%). There were no differences in socio-demographic characteristics for participants with and without postnatal depression except for household monthly income and women’s educational status. A higher proportion of study participants with depression had low income (60.2%) compared to those without depression (47.7%) (*p* = 0.08). Similarly, a higher proportion of those with depression was in the primary education category (39.8%) than those without depression (23.9%) (*p* = 0.015).

**Table 1:** Sociodemographic characteristics of study participants included in Gondar town, Ethiopia, 2018

Variable/category	Postnatal depression		Total (*n* = 895), n (%)	*p*-value
	Yes (*n* = 83), n (%)	No (*n* = 772), n (%)		
Women’s age at enrolment				0.495
18–24	23 (27.7)	259 (31.9)	282 (31.5)	
25–34	52 (62.7)	499 (61.5)	551 (61.6)	
> =35	8 (9.6)	54 (6.6)	62 (6.9)	
Mean (±SD)	26.7 (0.53)	26.5 (0.15)	26.5 (0.15)	
Household monthly income				0.080
Low	50 (60.2)	387 (47.7)	437 (48.8)	
Medium	27 (32.5)	338 (41.6)	365 (40.8)	
High	6 (7.3)	87 (10.7)	93 (10.4)	
Mean(±SD)	2920.5 (238.6)	3576.9 (107.0)	3516 (99.7)	
Women’s education				0.015
None	10 (12.0)	104 (12.8)	114 (12.7)	
Primary	33 (39.8)	194 (23.9)	227 (25.4)	
High school	26 (31.3)	313 (38.5)	339 (37.9)	
Tertiary	14 (16.9)	201 (24.8)	215 (24.0)	
Women’ occupation				0.224
Home duties	62 (74.7)	577 (71.1)	639 (71.4)	
Government employee	8 (9.6)	134 (16.5)	142 (15.9)	
Self-employee	13 (15.7)	101 (12.4)	114 (12.7)	
Women’s religion				0.761
Orthodox Christian	68 (81.9)	654 (80.5)	722 (80.7)	
Muslim	15 (18.1)	158 (19.5)	173 (19.3)	
Women’s marital status				0.250
Single	3 (3.6)	16 (2.0)	19 (2.1)	
Partnered	77 (92.8)	783 (96.4)	860 (96.1)	
Separated	3 (3.6)	13 (1.6)	16 (1.8)	
Difficulty accessing food in the last three months				0.360
Yes	5 (6.0)	32 (3.9)	37 (4.1)	
No	78 (94.0)	780 (96.1)	858 (95.9)	

Note: *p*-value was based on chi-square test statistics

### Obstetric and behavioral characteristics of study participants

The obstetric and behavioral aspects of the study participants are shown in Table 2. A higher incidence of postnatal depression was observed amongst the following participants: not using postnatal care services (*p* = 0.036), those that self-reported labor complications (*p* < 0.001), mothers whose babies had low birth weight (*p* < 0.001), and those with stillbirths (*p* < 0.001). Most of the pregnancies were planned (85.2%), and the mean (SD) number of children per woman was 2.1 (±1.2). Most participants (95.7%) had at least one antenatal care visit, 84.0% had a vaginal delivery, and 91.4% had no nutritional problem. Five-hundred and thirty-three participants (60.7%) initiated breastfeeding within an hour after birth, and 147 (16.4%) had preterm births.

**Table 2:** Obstetric and behavioral characteristics of study participants included in Gondar town, Ethiopia, 2018

Variable/category	Postnatal depression		Total *n* = 895, n (%)	*p*-value
	Yes (*n* = 83), n (%)	No (*n* = 772), n (%)		
Pregnancy intention				0.122
Planned	66 (79.5)	697 (85.8)	812 (85.2)	
Unplanned	17 (20.5)	115 (14.2)	83 (14.8)	
Parity of the mother				0.88
1	31 (37.4)	310 (38.2)	341 (38.1)	
2	25 (30.1)	259 (31.9)	284 (31.7)	
3–8	27 (32.5)	243 (29.9)	270 (30.2)	
Mean (±SD)	2.2 (1.2)	2.1 (1.2)	2.1 (1.2)	
Antenatal care service uptake (at least one)				0.78
Yes	79 (95.2)	778 (95.8)	857 (95.7)	
No	4 (4.8)	34 (4.2)	38 (4.3)	
Postnatal care service				0.036
Yes	48 (64.9)	624 (77.6)	672 (76.5)	
No	26 (35.1)	180 (22.4)	206 (23.5)	
Mode of delivery				0.58
Vaginal	63 (86.3)	675 (83.8)	738 (84.0)	
Cesarean section	10 (13.7)	130 (16.2)	140 (16.0)	
History of a self-reported labor complication				< 0.001
Yes	64 (77.1)	751 (92.6)	815 (91.2)	
No	19 (22.9)	60 (7.4)	79 (8.8)	
Early initiation of breastfeeding (BF)				0.183
Yes (≤ one hour)	49 (68.1)	484 (60.0)	533 (60.7)	
No	23 (31.9)	322 (40.0)	345 (39.3)	
Median hours at first initiation of BF (Median(±IQR))	1 (1–6)	1 (1–30)	1 (1–30)	
History of low birth weight				< 0.001
Yes	66 (79.5)	30 (3.7)	47 (5.3)	
No	17 (20.5)	782 (96.3)	848 (94.7)	
Postnatal nutritional status (MUAC)				0.95
Underweight (18–22)	7 (8.4)	70 (8.6)	77 (8.6)	
Normal (22.5–31)	76 (91.6)	742 (91.4)	818 (91.4)	
MUAC (Mean(±SD))	83 (0.08)	812 (0.07)	895 (0.09)	
History of preterm Birth				0.671
Yes	68 (81.9)	132 (16.3)	147 (16.4)	
No	15 (18.1)	680 (83.7)	748 (83.6)	
History of stillbirth				< 0.001
Yes	10 (12.1)	7 (0.9)	17 (1.9)	
No	73 (87.9)	805 (99.1)	878 (98.1)	

Note: *p*-value was based on chi-square test statistics

### Psycho-social characteristics of study participants

Table 3 shows the participants’ psycho-social characteristics. There were 76 (8.7%) study participants with poor marital relationships, 62 (6.9%) with a history of common mental disorders before pregnancy, 179 (20.0%) who had low social support, and 413 (47.1%) who reported frequent support from their partners. Study participants with postnatal depression more frequently reported a poor marital situation (*p* = 0.001), a history of a common mental disorder before pregnancy (*p* < 0.001), low partner support (*p* = 0.02), and depression during pregnancy (*p* < 0.001).

**Table 3:** Psycho-social characteristics of study participants included in Gondar town, Ethiopia, 2018

Variable/category	Postnatal depression		Total *n* = 895, n (%)	*p*-value
	Yes (*n* = 163), n (%)	No (*n* = 732), n (%)		
Marital situation (Women’s perspective)				0.001
Poor	12 (15.1)	64 (8.0)	76 (8.7)	
Good	61 (76.2)	527 (66.2)	588 (67.1)	
Very good	7 (8.7)	205 (25.8)	212 (24.2)	
History of common mental disorders				< 0.001
Yes	14 (16.9)	48 (5.9)	62 (6.9)	
No	69 (83.1)	764 (94.1)	833 (93.1)	
Social support				0.30
Poor	13 (15.7)	166 (20.4)	179 (20.0)	
Good	70 (84.3)	646 (79.6)	716 (80.0)	
Social support scale (Median (±IQR))	11 (9–13)	11 (9–13)	11 (9–13)	
Internal consistency (α)	0.76 (high reliability)			
Partner support				0.02
Always	29 (36.3)	384 (48.2)	413 (47.1)	
Most of the time	22 (27.5)	238 (29.9)	260 (29.7)	
Some of the time	25 (31.2)	139 (17.5)	164 (18.7)	
Rarely	4 (5.0)	35 (4.4)	39 (4.5)	
A symptom of antenatal depression				< 0.001
Yes	18 (21.7)	40 (4.9)	58	
No	65 (78.3)	772 (95.1)	837	
Depression scale (Median (±IQR))	6 (3–11)	4 (1–7)	4 (2–7)	
Internal consistency (α)	0.74 (High reliability)			

Note: *p*-value was based on chi-square test statistics

### Incidence and prevalence of postnatal depression

The initial screening for depression was conducted during pregnancy. Subsequently, a cohort comprising 895 women were rescreened for depression from two to six weeks post-delivery. Of 895 women rescreened, 83 (9.27%) screened positive for postnatal depression (95%CI: 7.45, 11.36). Sixty-five women developed symptoms of depression after their screening during pregnancy, indicating an incidence proportion of 7.77% (95%CI: 6.04, 9.79) and 18 (2.1%) women with protracted depression (positive for depression at both times of screening). Furthermore, 105 women had a depression in at least one of the screening times, giving a perinatal depression rate of 11.7%.

In the bivariate analyses, the prevalence of postnatal depression was higher among study participants who had low education and household income, did not access postnatal care services, reported labor complications, had low birth weight and stillbirth infants. Moreover, depression was also higher among study participants who reported poor marital relationships, poor husband support, and who had a history of common mental disorders before pregnancy and depression during pregnancy.

### Predictors of the incident and prevalent postnatal depression

Tables 4 and 5 shows predictors of incidence postnatal depression from a multivariable modified Poisson regression model. In the multivariable modified Poisson regression model, postnatal care service, history of common mental disorders before pregnancy, and depression score during pregnancy independently predicted the incidence of postnatal depression. The incidence risk of postnatal depression was 1.8 times higher (ARR, 95%CI: 1.0, 3.2) among study participants who did not access postnatal care service. An increase in one Edinburgh Postnatal Depression Scale (EPDS) score during pregnancy increased the risk of postnatal depression by 1.6 times (ARR, 95%CI: 1.4, 1.7). Women with a history of a CMD before pregnancy had 2.4 times higher (ARR, 95%CI: 1.4, 4.3) risk of postnatal depression than those who did not have CMD.

**Table 4:** Bi-variable and multivariable modified Poisson regression model of predictors of incidence of postnatal depression in Gondar town, Ethiopia, 2018

Variable	Postnatal depression (*N* = 895)		CRR^a^, 95%CI	ARR^b^, 95%CI
	Yes, N (%)	No, N (%)		
Postnatal care service (No)	17 (8.9)	174 (91.1)	1.2 (0.7, 2.0)	1.8 (1.0, 3.2)
Depression before pregnancy (Yes)	12 (23.1)	40 (76.9)	3.4 (1.9, 5.9)	2.4 (1.4, 4.3)
Antenatal EPDS score (median, IQR)	5 (2,7)	3 (1, 6)	1.4 (1.3, 1.5)	1.6 (1.4, 1.7)

^a^Crude relative risk. ^b^Adjusted relative risk; adjusted for educational status, monthly income, low birth weight, stillbirth, self-reported labor complication, pregnancy intention, early initiation of breastfeeding, marital agreement and husband support during pregnancy

**Table 5:** Direct, Indirect and total effect of stressors and mediators on postnatal depression among study participants (*N* = 895), Gondar town, Ethiopia, 2018

Risk factors	Direct effect β, SE	Indirect effect β, SE	Total effect β, SE
Antenatal depression			
Yes	0.29 (0.062) **	0.07 (0.036) *	0.36 (0.053) **
No	reference		
Low birth weight			
Yes	0.32 (0.064) **		0.32 (0.064) **
No	reference		
Self-reported labor complication			
Yes	0.09 (0.37) *	−0.004 (0.016)	0.087 (0.043) *
No	reference		
Postnatal care service			
Yes	−0.03 (0.089)		−0.03 (0.089)
No	reference		
History of CMD before pregnancy			
Yes	0.06 (0.032) *	0.05 (0.02) **	0.11 (0.03) **
No	reference		

** ≤ 0.001, * < 0.01. All β coefficients are standardized estimates

### Structural equation model

#### Confirmatory factor analysis for EPDS

**Fig. 2:**
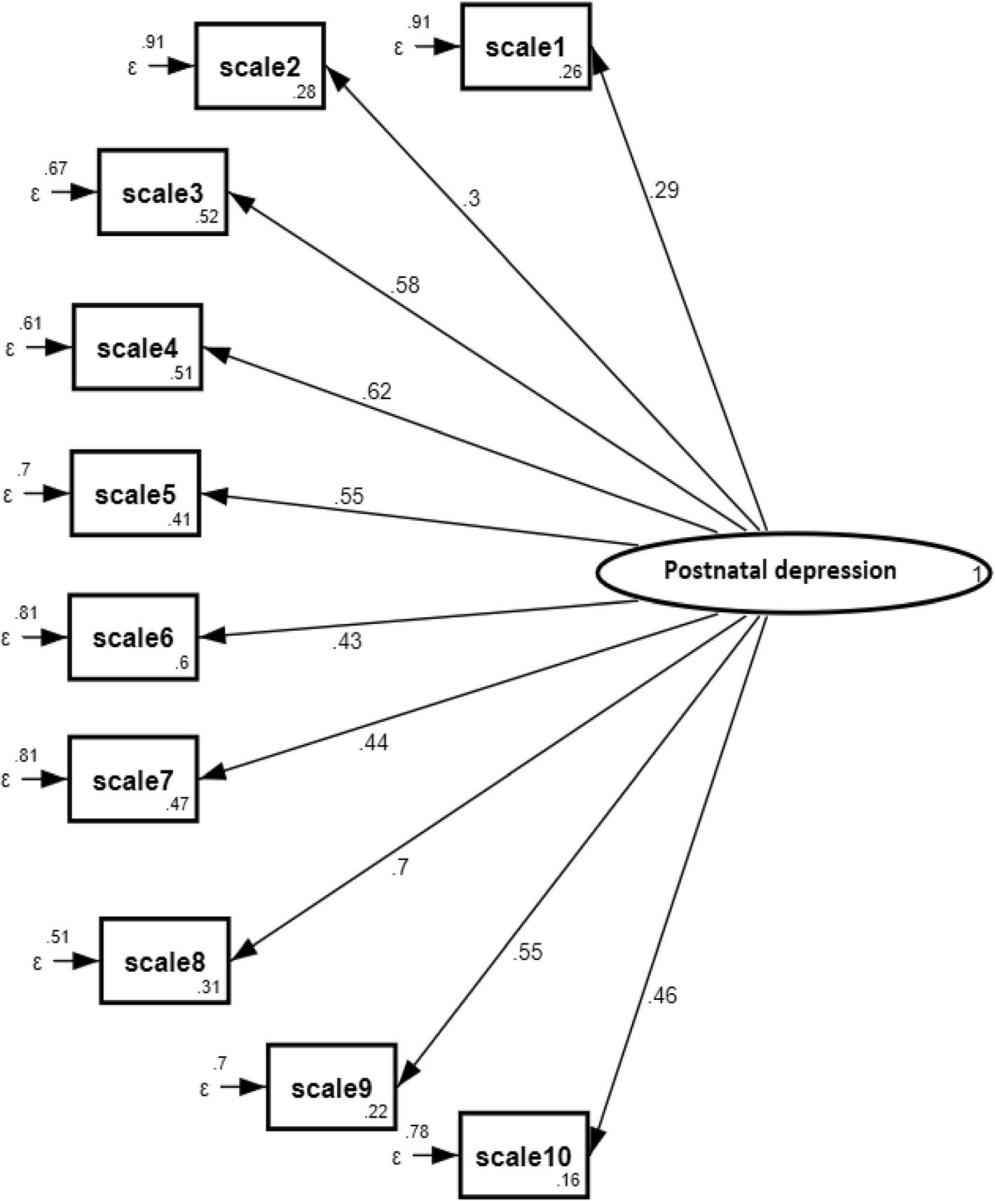
Results of a standardized factor loadings of a measurement model (items of EPDS) (*N* = 895), Gondar town, Ethiopia, 2018. Note: ** ≤ 0.001, * ≤ 0.05, β’s are standardized estimates

The model for postnatal depression had a satisfactory fit (CFI = 0.78, TLI = 0.71, SRMR = 0.06, R^2^ = 0.80) and all the factor loadings were significant at *p* < 0.001. The standardized factor loadings for each item are shown in Fig. 2.

#### Structural model

The model was adjusted for educational status, monthly income, stillbirth, pregnancy intention, early initiation of breastfeeding, marital situation, and husband support during pregnancy. After adjustment, postnatal depression symptoms were higher in those with self-reported labor complications (β = 0.86, *p* = 0.004), low birth weight (β = 0.85, *p* = 0.043), initiating breastfeeding early (β = 0.82, p < 0.001), not attending postnatal care services (β = 0.67, *p* = 0.005), those with symptoms of common mental disorders before pregnancy (β = 0.74, *p* = 0.013) and depression symptoms during pregnancy (β = 2.78, *p* < 0.001).

**Fig. 3:**
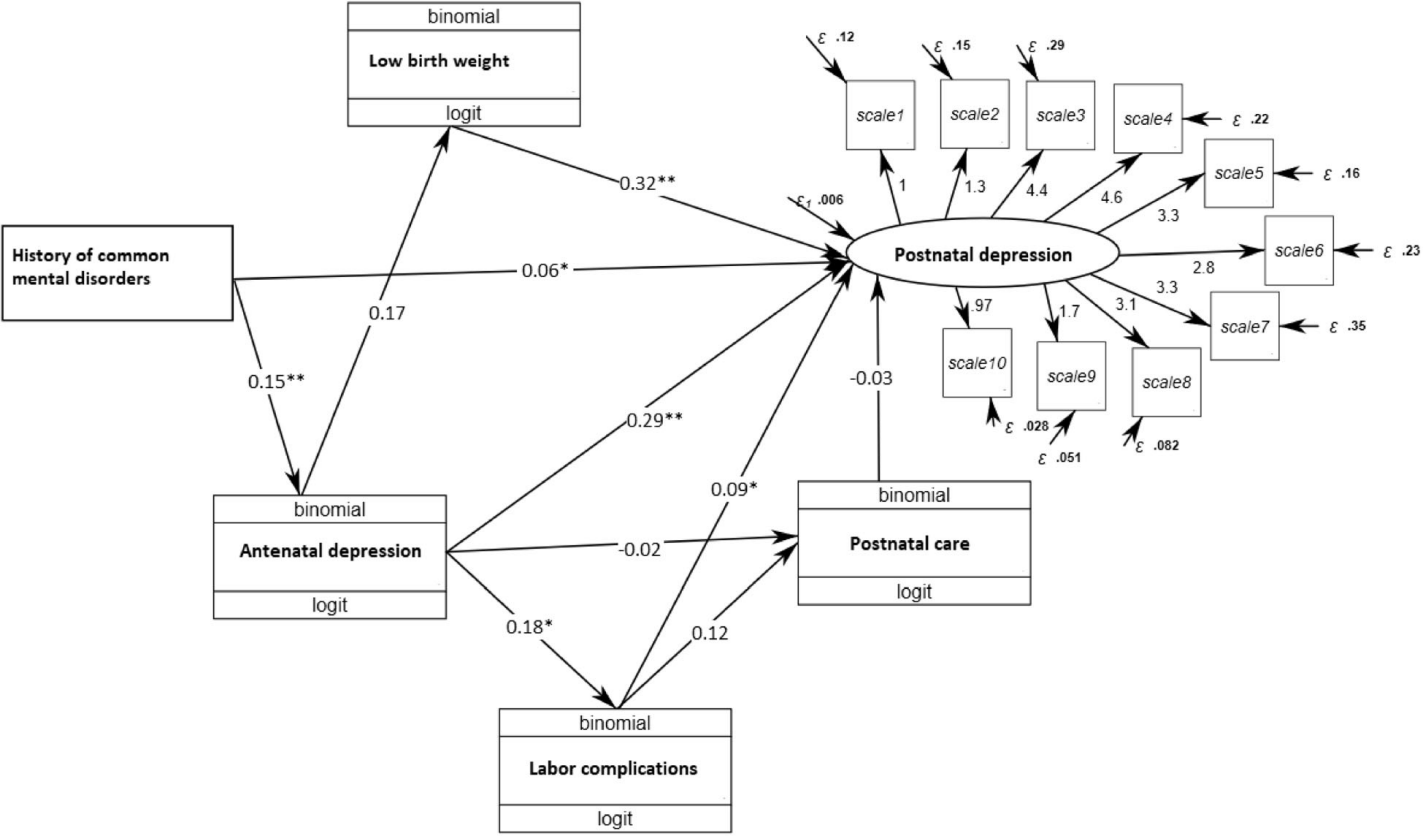
A generalized structure equation model of the stress-process model framework for postnatal depression using data from Gondar Town, Ethiopia. Note: ** ≤ 0.001, * ≤ 0.05, β’s are standardized estimates

Model fit for the GSEM was adequate according to the Mplus fit statistics (RMSEA = 0.062, CFI = 0.815, TLI = 0.772). Figure 3 shows the SEM for the EPDS according to the stress-process theoretical model. Based on the GSEM model, antenatal depression had both a direct (standardized β = 0.29) and indirect (standardized β = 0.07) effects on postnatal depression (total effect standardized β = 0.36). Thus, having antenatal depression was associated with 0.29 standard deviations (SD) higher postnatal depression and 0.36 SD higher score in the presence of labor complications.

Symptoms of common mental disorders (CMDs) before pregnancy were both directly (standardized β = 0.06) and indirectly (standardized β = 0.05) associated with the EPDS (total standardized β = 0.11). Thus, a history of CMDs was associated with an overall 0.11 SD higher EPDS. The two significant indirect pathways through which CMDs led to postnatal depression were through antenatal depression and self-reported labor complications. Low birth weight (standardized β = 0.32) and self-reported labor complications (standardized β = 0.09) also had direct positive associations with postnatal depression. Having a LBW infant and a self-reported labor complication were associated with a 0.32 and 0.09 SD higher EPDS respectively.

## Discussion

In low-income countries, postnatal depression is one of the most under-investigated and under-recognized common mental health disorders, yet nonetheless represents a severe threat to the wellbeing of mothers and their newborn babies. Although postnatal depression affects nearly a quarter of postnatal women worldwide, its complications and manifestations are often overlooked as being symptoms of less severe postnatal blues or psychosis [56, 57]. In Ethiopia, postnatal depression is not well recognized and studied as part of the perinatal continuum of care and lacks government priority. In this community-based cohort study, pregnant women were screened before and after child delivery to explore the extent of, and development of, depression during the perinatal period and to identify risk factors for postnatal depression. The stress–process model was used as the theoretical framework to test and identify the most likely sources of postnatal depression stressors and their interrelationships. In this study, the prevalence and incidence of PND were 9.3 and 7.8%, respectively, and 2.1% of the women had persistent depression. PND was independently predicted by having no postnatal care services, having Antenatal Depression (AND) and Common Mental Disorders (CMD) before pregnancy. Antenatal depression and a CMD before pregnancy had both a direct and an indirect positive effect on PND scores. Low birth weight (LBW) and self-reported labor complications had direct effects only on PND scores.

In this study, 11.7 and 9.3% of women reported symptoms of perinatal and postnatal depression respectively, consistent with a pooled estimate of postnatal depression in Ethiopia and a study conducted in Sudan (9.2%) [58]. However, the prevalence of postnatal depression was far lower than that estimated in a community-based cohort study conducted in the Sodo district (22.1%) [25]. Similarly, our current findings suggest lower rates than those estimated for the community based cross-sectional studies conducted in Mizan Aman town (22.4%) [28] and Sodo district (12.7%) [24] as well as the health institution-based cross-sectional studies conducted in Addis Ababa (22.3%) [26] and South West Ethiopia (33.8%) [26]. However, these studies differ from the current study regarding the depression screening tools that were used, study design and setting. Each used different tools (PHQ and SRQ) to measure postnatal depression and these have different sensitivities and specificities to the EPDS. Further, except for the two studies conducted in the Sodo district [24, 25], the studies defined postnatal depression irrespective of the time since delivery, thereby potentially over-estimating the prevalence since mild depression and mood swings (baby blues) are very common in the first two weeks after delivery [59–61].

Previous studies were institutional-based and cross-sectional in design, while we used a community-based cohort design. In Ethiopia, relatively few postnatal mothers attend health facilities for postnatal care [62]. Those access these health facilities might be more likely to report the presence of mental health issues due to better awareness about postnatal care or due to experiencing health conditions (in themselves or their newborn) that require medical consultation. The current study estimate for depression prevalence is slightly higher than a rural-based study conducted in Ethiopia (4.6%) [63] and Ghana (3.8%) [64]. The variation in prevalence might be due to the time of the investigation, tools used for screening, and protocol-specific differences.

The incidence proportion of postnatal depression was 7.8%, of which 2.1% of women had depression during and after pregnancy (persistent depression). The incidence proportion of persistent depression was much lower than that observed in a prospective community-based study conducted in a rural area of Ethiopia [25], which reported an incidence of 15.4% postnatal depression and 11.1% persistent depression. The rural nature of the study, as well as the use of different tools, might explain this higher incidence. In a review of longitudinal studies, 7% of women reported persistent depressive symptoms in pregnancy and during the postnatal period [65]. Since the current study excluded women at higher risk of prenatal and postnatal depression for ethical reasons, this exclusion has likely lowered our results for persistent depression. However, our results were higher than the incidence (2.4%) and persistence (2.5%) of common mental health disorders found in Butajira, Ethiopia [63].

Perinatal depression was lower than previous cohort studies in rural Ethiopia [25, 63] and consistent with a review of longitudinal studies on perinatal depression [65]. Given the negative impact of PND on infant health and developmental consequences [66–68], these results emphasize the need for support driven from the federal ministry of health of the government of Ethiopia to address perinatal depression.

Symptoms of common mental disorders (CMDs) before pregnancy and depression during pregnancy were predictors of postnatal depression symptoms. The risk of postnatal depression increased by 1.6 and 2.4 times for women who have had antenatal depression and CMDs before pregnancy, respectively. In the path analysis, CMDs before pregnancy and depression during pregnancy both had direct and indirect effects on postnatal depression. Besides, antenatal depression mediated the association between CMDs before pregnancy and postnatal depression. A strong association between previous depressive symptoms and postnatal depression has previously been reported in low [28, 64] and high-income countries and systematic reviews [21, 59, 65, 69]. This highlights how depression can persist or re-occur during pregnancy. Early screening and intervention are therefore important to prevent such complications and poor obstetric and perinatal outcomes as well as the health effects on newborn and its development [70, 71].

Biological and environmental explanations for depression during the perinatal period include psycho-social and economic stressors that can affect the normal functioning of the hypothalamic-pituitary-adrenal axis activity prior to pregnancy and continue during pregnancy and after birth [72]. Increased placental cortisol hormone production can cause persistent symptoms of depression during pregnancy, whilst sharp reductions in cortisol hormone production following the birth can cause depressive symptoms to develop postnatally [73–75]. Public health and clinical interventions include the screening and treating of depression symptoms during pregnancy and if possible pre-pregnancy preparation [76].

In the current study, the use of postnatal care service (PNC) decreased the risk of incident postnatal depression. Postnatal depression was increased by 80% in women who had no PNC service. Previous studies conducted on postnatal depression in Ethiopia [24–28, 63] and in other African countries [58, 77–82] did not adjust for PNC. Postnatal care services that assess the emotional health of the pregnant women might, therefore, help prevent post-natal depression. Low birth weight (LBW) was directly and positively associated with postnatal depression. Previous studies conducted outside of Ethiopia have also reported a positive association between LBW and postnatal depression. In China, LBW was indirectly associated with postnatal depression [32] and this finding has also been replicated in developed countries [83, 84]. In the systematic review by Vigor et al., mothers of LBW or preterm infants had sustained depression for one year postpartum compared to normal weight mothers [15]. It has been reported that experiencing LBW can pose a challenge interfering with an early adaptation of parenthood, parental roles, and leads to a severe strain that negatively affects a mother’s caring practice [85].

Low breastfeeding self-efficacy was a significant predictor of postnatal depression in the Avon Longitudinal Study of Parents and Children [86], whilst in Iran, breastfeeding self-efficacy training in postnatal women with LBW significantly improved maternal stress and depression [87]. In the current study, early initiation of breastfeeding was associated with an increased postnatal depression score. However, the association was not significant in the path model. The effect of breastfeeding on postnatal depression is highly heterogeneous and is mediated by both maternal breastfeeding intention and mental wellbeing during pregnancy [88]. Possible explanations for these findings include postnatal mothers with labor complications or that have LBW or preterm birth infants being more likely to stay in hospitals, which generally encourage early breastfeeding. There is also the possibility that the initiation of breastfeeding may cause pain and increase stress. Further exploration is required to identify why the early initiation of breastfeeding was associated with an increased postnatal depression score.

Similar to a study from the Sodo district in Ethiopia [24], self-reported labor complications were directly associated with postnatal depression, but the effect was small. Similarly, Weobong reported a high risk of postnatal depression among women with long labor times [64] and an Indian study [89], a Nepalese study [90], and a systematic review reported negative birth experiences such as elevated labor pain as risk factors for postnatal depression [13]. Childbirth processes might trigger painful memories and women who previously experienced difficult labor, or complicated pregnancies have a higher likelihood of developing Post-traumatic Stress Disorders (PTD), which might lead to depression symptoms [91]. Furthermore, because of high maternal mortality in Ethiopia, such complications are more likely to be perceived as life-threatening and could potentially affect the mental wellbeing of the postnatal mothers [92]. Attention should, therefore, be given to mothers with a history of labor complications in order to intervene early to address the increased risk of postnatal depression.

### Strength, limitation, and public health implications

To our knowledge, this is the first study to explore the stress pathways leading to postnatal depression, applying a stress-process theoretical framework in an urban region in Ethiopia. Through a structural equation modelling frame of analysis, a conceptual framework on the relationship between risk factors for postnatal depression was developed with adequate sample size and a low attrition rate. This approach allowed us to simultaneously examine the direct and indirect associations between various hypothesized risk factors and postnatal depression. The study had several limitations. Firstly, self-reports were used to assess previous exposures to depression, and this might have led to recall bias. Second, assessment of labor complications was based on the mother’s evaluation, which may have introduced inaccuracies, increasing or reducing the associations depending on personal perceptions. Third, the use of a screening tool solely to measure depression could have resulted in misclassification of women with and without depression. Fourth, the exclusion of women with a high possibility of depression could underestimate the true burden in the community. Finally, other important factors, such as experiences of violence, were not measured, and this could lead to unobserved confounding.

Although the prevalence of postnatal depression in this study was relatively low, its impact on child development, women and the economy was highly significant, particularly in countries with large populations and low income. For example; economic analysis of perinatal depression in Australia in 2013 showed that not treating perinatal depression cost the economy AUD$538 M [93]. Ethiopia has a population four times larger with a fertility rate nearly three times as high, which represent a potential total coast of approximately $4 billion. Moreover, a recent study on catastrophic health expenditure in rural Ethiopia found high out-of-pocket payments at the point of service for depression-related reasons which could also be expected to be much higher in urban areas [94].

## Conclusions

Incidence and prevalence of postnatal depression found in this study were lower than the previously published findings from cross-sectional studies in Ethiopia. Mental health disorders before pregnancy and depression during pregnancy were found to be important predictors of incident postnatal depression. Both also had a direct and indirect effect on prevalent postnatal depression. Depression during pregnancy mediated the causal link between common mental disorders before pregnancy and prevalent postnatal depression. Postnatal care service predicted the risk of incident postnatal depression. Self-reported labor complication and low birth weight were associated with prevalent postnatal depression while self-reported labor complications mediated the link between antenatal depression and postnatal depression. Early detection and management of depression during the prenatal period would prevent its maternal complications, postnatal depression, and child developmental consequences.

## Abbreviations

EPDS: Edinburgh Postnatal Depression Scale
RR: Relative Risk
WHO: World Health Organization
LBW: Low birth weight
CMD: Common Mental Disorder
ODK: Open Data Collection Kit
LNMP: last normal menstrual period
ANC: Antenatal care
OSSS: Oslo Social Support Scale
MUAC: Middle-Upper Arm Circumference
PCI-4: Perinatal Coping Inventory
GSEM: Generalized Structural Equation Modeling
SD: Standard deviation
